# Effect of testosterone on blood-clotting markers in transsexual men

**DOI:** 10.1016/j.htct.2025.103862

**Published:** 2025-06-14

**Authors:** Estella Thaisa Sontag dos Reis, Carla Maria Franco Dias, Carolina Sales Vieira, Mariane Nunes Nadai, Sérgio Henrique Pires Okano, Silvio Antônio Franceschini, Lúcia Alves da Silva Lara

**Affiliations:** Faculdade de Medicina de Ribeirão Preto da Universidade de São Paulo (FMRP-USP), Ribeirão Preto, SP, Brazil

**Keywords:** Testosterone, Transsexual men, Hormone therapy, Coagulation

## Abstract

**Background:**

The use of testosterone in gender-affirming hormone therapy for trans men is associated with several adverse effects. However, research on the risk of venous thromboembolism in this treatment remains limited and inconclusive. This study aimed to assess the impact of intramuscular testosterone on specific direct and indirect blood-clotting markers in trans men.

**Method:**

Treatment of trans men without previous use of testosterone was followed up in a prospective observational study in a trans people healthcare service. Gender-affirming hormone therapy was initiated with intramuscular testosterone cypionate (Depo-Testosterone). The blood-clotting markers prothrombin time, activated partial thromboplastin time, d-dimer, antithrombin, and factors VIII and VII were evaluated before and 12 weeks after starting the medication.

**Results:**

Nineteen trans men with a mean age of 23.7 ± 3.7 years were enrolled. After 12 weeks of hormone therapy, significant increases in weight (*p*-value = 0.002) and body mass index (*p*-value = 0.007) were observed in patients. Furthermore, there were significant increases of 830 % in serum testosterone (*p*-value = 0.000), 7 % in hemoglobin (*p*-value = 0.000) and 10 % in hematocrit (*p*-value = 0.001). Conversely, a 10 % decrease in high density lipoprotein cholesterol levels (*p*-value = 0.000), and 15 % decrease in Factor VII (*p*-value = 0.000) were detected.

**Conclusion:**

Intramuscular testosterone in trans men was associated with increases in hematocrit, hemoglobin, and the body mass index, and decreases in high density lipoprotein cholesterol and Factor VII. Nevertheless, these variables remained within normal reference values. Long-term follow-up studies evaluating gender-affirming hormone therapy with testosterone are needed to determine adequate risk management of venous and arterial thromboembolism in this population.

## Introduction

Gender-affirming hormone therapy (GAHT) for transgender men involves the administration of exogenous testosterone to promote the development of standard male characteristics. Desired characteristics include increased body and facial hair, deepening of the voice, increased muscle mass and reduced fat mass, as well as blocking the menstrual cycle and enlarging the clitoris.^1^ In the laboratory setting, GAHT for trans men aims to elevate serum testosterone and reduce estradiol concentrations to achieve masculinizing levels.^2^ Although these medications are considered relatively safe in the short and medium term,^3^ GAHT is associated with adverse effects that may pose risks to the health of this population.^4^

The most common adverse effects attributable to testosterone use include erythrocytosis,^5^ acne,^6^ hair loss, increased sexual desire, temporary or permanent reduction in fertility,^2^ and alterations in the lipid profile, with a decline in high-density lipoprotein cholesterol (HDL-c) and increases in both triglycerides (TG) and low-density lipoprotein cholesterol (LDL-c).^4^ There is also a reduction in adiponectin levels associated with insulin resistance, with a greater predisposition to type II diabetes in this population.^7^ Another concern is the increased risk of thromboembolism associated with testosterone use. However, research on this topic is limited in the literature, especially in the trans men population.^8^^,^^9^

The largest study available in the literature showed that the hazard ratio of venous thromboembolism (VTE) in trans men compared to cisgender (*cis*) men was 1.6 (95 % confidence interval [95 % CI]: 0.9–2.9) in the total group and 2.7 (95 % CI: 0.6–12.1) in the group starting GAHT with testosterone.^10^ Although the observed increases in the risk of VTE from 60 % to 270 % were not statistically significant,^10^ they may hold clinical relevance, especially in this population, which presents other risk factors for VTE, such as a higher prevalence of smoking and erythrocytosis.^11^ It is important to highlight that the number of trans men that had VTE in these studies was considerably small, a fact that makes it difficult to draw an adequate conclusion regarding the risk of VTE in transgender men.^9^

In the absence of studies with a suitable sample size to reach a definitive conclusion about the risk of VTE in trans men, the effects of testosterone on blood clotting may be useful for generating hypotheses regarding the role of testosterone in hemostasis and in the risk of VTE. A recent study analyzed fibrinogen, specific blood clotting factors (FII, FIX, and FXI), natural anticoagulants (protein S and protein C), resistance to activated protein C, and hematocrit in trans men.^8^ According to the authors, the use of testosterone, regardless of the route of administration (transdermal or intramuscular) for 12 months was not associated with relevant procoagulant alterations.^8^ However, it is important to note that some clotting markers have not been evaluated in trans men using testosterone.

Given the lack of conclusive information regarding the risk of VTE and the paucity of studies on the effects of testosterone on hemostasis in this population, the aim of the present study was to assess the impact of intramuscular testosterone use on the hemostatic system of trans men 12 weeks after initiating GAHT through the quantification of blood-clotting markers.

## Methods

### Study design and settings

This study employed a prospective observational cohort design, enrolling trans men without previous use of testosterone, and was carried out in a trans people healthcare service at the Clinics Hospital and the “Saúde Escola” Center of Ribeirão Preto Medical School, University of São Paulo, Brazil. The study participants were selected by convenience sampling, enrolling all eligible patients who wished to start GAHT with testosterone. The studied period was from December 2021 to December 2022.

### Compliance with ethical standards

The present research project was approved by the Research Ethics Committee of the Clinics Hospital of the Ribeirão Preto Medical School. The study was conducted in accordance with the principles of the Declaration of Helsinki. Informed consent was obtained from all participants included in the study.

### Participants

The participants were recruited by the main researcher (ETSR), who contacted the participants prior to the start of GAHT, explained the objectives of the study, and invited them to participate. Individuals aged from 18 to 40 years, with female genitalia and male gender identity, and who desired to initiate GAHT with testosterone were considered eligible for the study. The exclusion criteria were: smoking, history of current or previous arterial or venous thromboembolic disease, body mass index (BMI) ≥30 kg/m², contraindication to testosterone use, withdrawal from participating in the study or discontinuation of treatment.

### Methodology and variables

After informed consent, the recruited patients attended an initial medical evaluation before starting GAHT. During this visit, the researchers collected sociodemographic and clinical data and measured the participants’ weight and height to determine their body mass index (BMI).

A total of 20 mL of fasting venous blood was collected to measure the clotting times (prothrombin time - PT; International Normalized Ratio - INR; and activated partial thromboplastin time - APTT), clotting markers (activity of coagulation factors VII and VIII), the fibrin turnover marker (D-dimer), and the anticoagulation marker (antithrombin), as well as tests to monitor GAHT safety (lipid profile, hemogram, total testosterone, and estradiol).

After the initial evaluation, intramuscular testosterone cypionate (Depo-Testosterone) was prescribed every 21 days. To minimize and detect potential losses to follow-up, the participants were contacted biweekly by phone to ensure proper testosterone usage and to remind them about the tests scheduled for 12 weeks after starting GAHT.

The enzymatic method was adopted to measure total cholesterol (TC), HDL-c, LDL-c, and TG levels. The LDL-c was calculated using the Friedewald formula: LDL-*c* = TC - (HDL-*c* + TG/5), provided that the values were <400 mg/dL.^12^ Testosterone and estradiol levels were measured using the radioimmunoassay method.^13^

Meanwhile, in order to assess the clotting factors, venous blood was collected in tubes containing sodium citrate and centrifuged for 15 min at room temperature for platelet removal. The plasma supernatant was stored at −35 °C in 0.2-mL aliquots for later analyses, at which time the frozen plasma samples were thawed directly in a water bath at 37 °C for at least 15 min and mixed by shaking before use.

The PT was determined using the photo-optical method with the STA-Neoplastine CI reagent.^14^ The INR was calculated using two major PT ‘correction factors’, the mean normal PT and the international sensitivity index.^15^ The APTT was also determined using the photo-optical method, but with the STA-PTT reagent,^16^ which establishes the partial time of thromboplastin activation. This method is used to analyze clotting factors XII, XI, IX, X, V, II, and I.

Factor VIII was assessed by coagulometry using the STA-ImmunoDef VIII reagent.^14^ Factor VII was also measured through coagulometry, using an automated coagulometer (Instrumentation Laboratory, United Kingdom).^14^ Additionally, plasminogen activator inhibitor was evaluated using a chromogenic substrate assay.^14^

Antithrombin was measured using the chromogenic method by way of STA-Stchrom ATIII cleavage.^14^ The determination of d-dimer levels was conducted via immunoturbidimetry using the Imubind Dimer Test Stripwell EIA Kit.^17^

### Statistical analyses

Statistical analyses were conducted using the R software, version 4.2.2 (R Foundation for Statistical Computing), with the significance level set at *p*-value <0.05. Quantitative variables are summarized using measures of central tendency and dispersion. In order to detect possible statistical differences regarding the quantitative variables, the Wilcoxon non-parametric test for paired samples was used, as the variables did not present parametric distribution.

## Results

**Figure 1:**
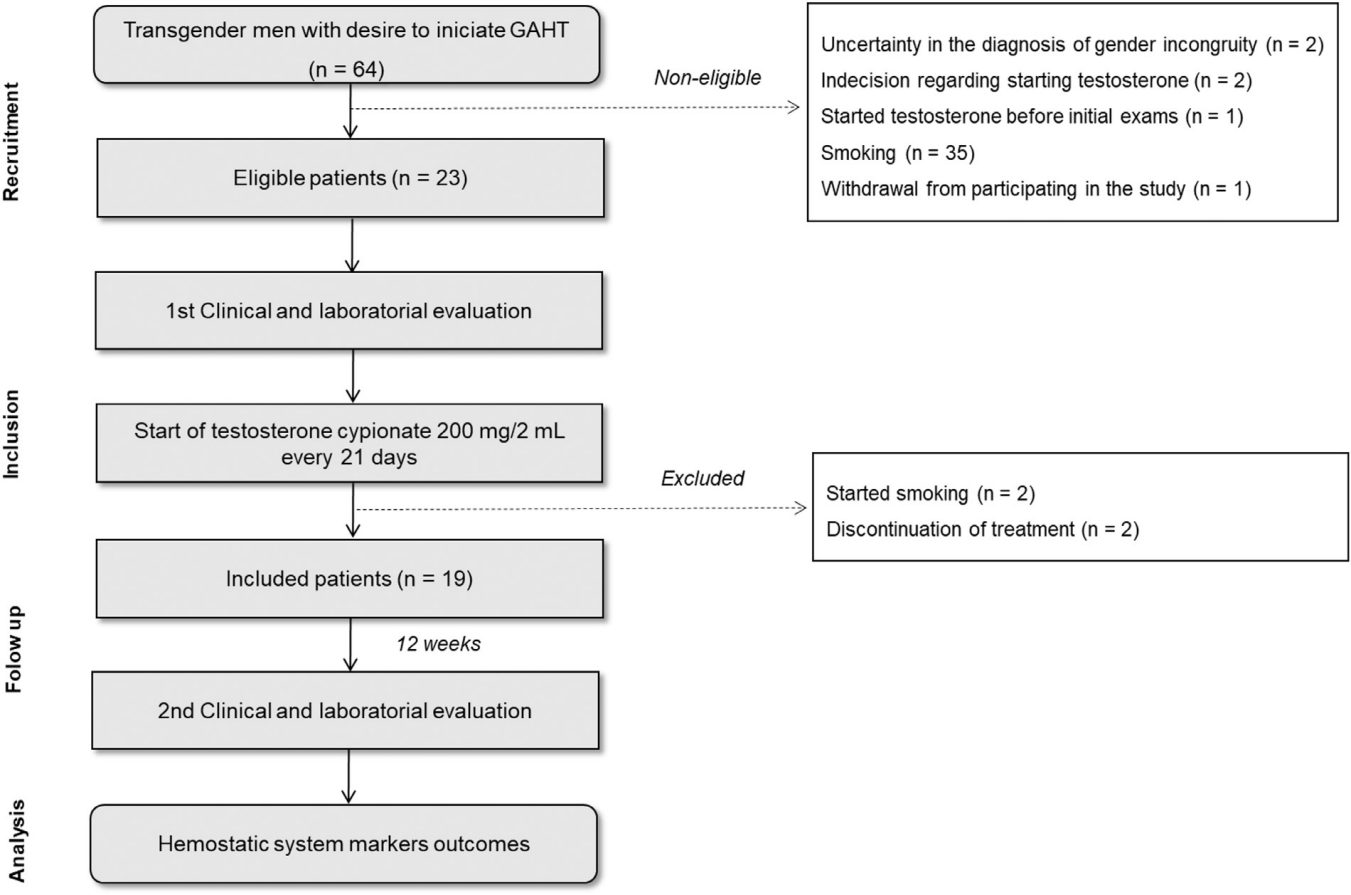
Flowchart of the study.

Sixty-four patients were recruited from December 2021 to December 2022. In the initial evaluation before starting GAHT, 41 patients were excluded from the study for the following reasons: uncertainty in the diagnosis of gender incongruity (*n* = 2); indecision regarding starting testosterone treatment (*n* = 2); previous use of testosterone (*n* = 1); smoking (*n* = 35); and withdrawal from participating in the study (*n* = 1). Thus, 23 patients were considered eligible and consented to participate. After the beginning of GAHT, four patients were excluded based on the following criteria: initiation of smoking (*n* = 2), and discontinuation of testosterone on their own (*n* = 2). Finally, 19 patients were included in the study and had a second clinical evaluation and blood collection 12 weeks after starting testosterone cypionate (Depo-Testosterone) treatment (Figure 1).

The mean age (± standard deviation) of the participants was 23.7 ± 3.7 years. Of the participants included in the study, five reported having comorbidities, including one case of type I diabetes mellitus and hypothyroidism (under use of insulin and levothyroxine (Levoxyl)), one case of dyslipidemia (without medication use), and two cases of depressive mood disorder taking psychoactive medications (sertraline, risperidone (Risperdal), and venlafaxine). In terms of contraceptive use, three participants were using intramuscular depot medroxyprogesterone acetate (Provera) every three months and one participant was using a copper intrauterine dispositive. None of the participants were using combined hormonal contraceptives.

The clinical and laboratory characteristics of the study participants are shown in Table 1. After 12 weeks of testosterone cypionate (Depo-Testosterone) use, there was a body weight gain of approximately 5 % (*p*-value = 0.002). An increase of 830 % was also observed in serum testosterone levels (p-value <0.001), as well as a 27 % reduction in estradiol (p-value = 0.08), and reduction in HDL-c, of around 10 % (*p*-value <0.001).

**Table 1:** Clinical and laboratory characteristics of transgender men before and 12 weeks after starting gender-affirming hormone therapy with testosterone.

Variable	Basal (Mean ± SD)		12 weeks (Mean ± SD)	*p*-value^a^
Age (years)	23.7 ± 3.74		n/a	
Weight (kg)	60.7 ± 9.16		63.62 ± 8.5	0.002
Height (m)	1.63 ± 0.05		n/a	
BMI (kg/m²)	22.9 ± 3.7		23.94 ± 3.6	0.007
Total cholesterol (mg/dL)	166.1 ± 33.7		163.9 ± 29.3	0.30
HDL (mg/dL)	51.9 ± 14.0		46.3 ± 11.2	0.006
LDL (mg/dL)	97.3 ± 33.7		99.6 ± 28.5	0.71
Triglycerides (mg/dL)	84.9 ± 37.7		89.4 ± 49.5	0.86
Testosterone (ng/dL)	58.2 ± 28.7		483.6 ± 324.6	<0.001
Estradiol (pg/mL)	78.0 ± 61.9		57.3 ± 47.8	0.08

SD: standard deviation; HDL: high-density lipoproteins; LDL: low-density lipoproteins; n/a: not applicable.

a Wilcoxon non-parametric test.

Table 2 shows the levels of blood-clotting markers before and after 12 weeks of GAHT with testosterone. Notably, there was a 7 % increase in hemoglobin (*p*-value <0.001), a 10 % rise in the hematocrit (p-value = 0.001), and a 5 % increase in INR (*p*-value = 0.046). A 15 % reduction in clotting factor VII was also observed (*p*-value <0.001), while the remaining variables showed no significant changes.

**Table 2:** Laboratory evaluation of the hemogram and hemostatic system markers of transgender men before and after gender-affirming hormone therapy with testosterone.

Variable	Basal (Mean ± SD)	12 weeks (Mean ± SD)	*p*-value^a^
Hemoglobin (g/dL)	13.8 ± 1.3	14.9 ± 1.6	<0.001
Hematocrit (%)	42.0 ± 4.1	46.2 ± 4.9	0.001
Platelets (x10³/µL)	289.4 ± 67.5	278.8 ± 69.8	0.55
D-dimer (μg/L)	0.3 ± 0.2	0.2 ± 0.1	0.31
Factor VII (%)	73.5 ± 23.5	62.4 ± 15.8	<0.001
Factor VIII (%)	127.7 ± 43.7	122.9 ± 36.3	0.65
Antithrombin (%)	106.7 ± 9.6	108.0 ± 7.8	0.43
APTT (seconds)	30.74 ± 3.24	32.37 ± 5.36	0.1183
PT (seconds)	12.06 ± 1.0	12.34 ± 1.09	0.1162
INR	1.00 ± 0.08	1.05 ± 0.13	0.046

SD: standard deviation; INR: International Normalized Ratio; APTT: activated partial thromboplastin time; PT: prothrombin time.

Reference values: d-Dimer: ≤0.5 μg/L; Factor VII: 50–129 %; Factor VIII: 50–150 %; Antithrombin: 80–120 %.

a Wilcoxon non-parametric test.

## Discussion

After 12 weeks of GAHT, a statistically significant reduction in clotting factor VII activity was observed, as well as statistically significant increases in hemoglobin and hematocrit. Nonetheless, these alterations remained within normal values. Meanwhile, the remaining markers of the hemostatic system did not show significant changes.

The activation of blood coagulation initiates with the formation of a complex between the tissue factor (TF) and the activated factor VII (FVIIa) composed of a serin protease with procoagulant properties.^18^ The 15 % reduction in factor VII activity, a deficiency which predisposes to the risk of hemorrhagic disorders,^18^ may contribute to an anticoagulant effect. In line with this hypothesis, a recent study evaluating blood clotting in 100 trans men before and after 12 months of GAHT evidenced an increase in factor IX activity and in the hematocrit, suggesting procoagulant alterations.^8^ On the other hand, in the same study, there were reductions in factor II and factor XI activity, in addition to increased levels of natural anticoagulants (protein S and activated protein C), which may have counterbalanced the procoagulant effect.^8^ Prospective studies could be designed to evaluate whether there is a correlation between reduced coagulation factors and erythrocytosis in trans men.

The relationship between coagulation factor VII deficiency,^19^ the activity of factors II, V, and X, and fibrinogen and the prolongation of the INR has already been documented in the literature.^15^ The PT is a single-stage screening test used to assess the TF and overall coagulation that is influenced by the activity of coagulation factors (II, V, VII, X) and fibrinogen.^15^ The prolongation of PT can be caused by deficiencies in one or more coagulation factors or may indicate the presence of coagulation factor inhibitors.^15^ Corroborating a previous report^1^ in which the use of testosterone in trans men was analyzed for over one year of follow-up, the present study observed an increase of 5 % in INR. However, no significant change in PT values was noted.

Among the possible procoagulant effects observed in this study, there was a 10 % increment in the hematocrit after 12 weeks of testosterone use. An elevated hematocrit indicates an increase in blood viscosity.^20^ This alteration could potentially reduce the venous return and elevate the cardiovascular risk; however, due to the lack of specific studies in this population, the parameters used refer to cisgender individuals. In one study involving *cis* men, a 5 % increment in the hematocrit increased the risk of VTE by 33 % (odds ratio: 1.33; 95 % CI: 1.05–1.70),^20^ while in *cis* women, this increase in hematocrit was not associated with increased risk when adjusted for age, BMI, and smoking status. It is noteworthy that trans men are at greater risk of elevated hematocrit due to the high prevalence of smoking.^21^ On the other hand, the actual relationship between erythrocytosis and VTE in the general population is still debated in the scientific literature,^8^ and there is still not enough data to determine whether or not erythrocytosis resulting from GAHT contributes to an increased risk of VTE in trans men.^22^

A systematic review conducted in 2021 showed that the incidence of VTE in trans men using testosterone was 10.8 in every 10,000 patients per year.^9^ This frequency is comparable to the rates seen in cisgender men undergoing hormone replacement therapy with testosterone. According to the authors of that review, the majority of current findings do not support an association between GAHT and testosterone and an increased risk of VTE.^9^

In the present study, regarding the lipid profile, after 12 weeks of starting GAHT with testosterone, a nearly 10 % reduction in HDL-c was observed in relation to the mean of the participants (*p*-value = 0.006). Conversely, the other lipid profile parameters, such as total cholesterol, LDL-c, and TG, did not show marked changes. This decrease in HDL-c is in line with a study carried out in 2017^23^ that investigated the effects of GAHT with testosterone on the lipid profile of trans men, in which the authors noted a reduction in HDL-c and an increase in LDL-c and in TG levels. However, the mechanism by which testosterone negatively impacts the lipid profile remains unknown.^24^ During the 12-week period, there was also an increase in body weight, on average, of approximately 3 kg among the participants and, consequently, an increase in BMI. The reduction of 1 mg/dL of HDL-c is correlated with an increase of 2–3 % in cardiovascular incidents.^25^^,^^26^

Together with data from the literature, the findings of this study point to the hypothesis that the use of GAHT is not associated with procoagulant alterations. Therefore, it does not seem to be through altering hemostasis that GAHT increases the risk of VTE. Further research is necessary to determine whether increments greater than 5 % in the hematocrit of trans men undergoing GAHT would be capable of increasing the risk of VTE due to elevated blood viscosity. Regarding arterial thrombosis, one study reported a 3.7-fold increase in the risk of acute myocardial infarction in trans men compared to *cis* women.^27^ The changes in the lipid profile caused by GAHT, with the reduction of HDL-c, may be implicated in this risk.

The present study should be interpreted in light of some limitations, namely the limited number of participants, the lack of an untreated control group, its observational design, and the short observation period. However, if even in small studies we have still not found a sign that GAHT can cause procoagulant alterations, the question arises as to what is the advantage of using more financial resources for randomized and controlled studies to evaluate this hypothesis. The exclusion of smokers in the present study represents a limitation, as it restricts the generalizability of the findings to the broader population of trans men undergoing GAHT. The high prevalence of smoking in the trans population has already been documented in the literature. When compared to their cisgender counterparts, transgender individuals are 2.7 times more likely to consume cigarettes throughout their lives.^28^ Future clinical studies should consider including participants with diverse clinical profiles, including individuals who smoke, to better capture the variability of hemostatic responses. Additionally, expanding the sample to include trans men in different clinical conditions would provide a more comprehensive understanding of the potential cardiovascular and hemostatic effects of hormone therapy across heterogeneous populations.

## Conclusion

GAHT with testosterone in 12 weeks of observation promoted an increase in hematocrit, hemoglobin, weight, and BMI, as well as a reduction in HDL-c and clotting factor VII; however, these alterations remained within the normal limits for the variables analyzed. Considering the increased hematocrit and the reduced HDL-c, it might be more pertinent to invest in studies that evaluate risk factors for arterial thrombosis in trans men. Finally, long-term follow-up studies of trans individuals are necessary to determine the actual risk of venous and arterial thromboembolism in this population and provide adequate risk management after the start of GAHT.

## CRediT authorship contribution statement

**Estella Thaisa Sontag dos Reis:** Investigation, Formal analysis, Writing – original draft. **Carla Maria Franco Dias:** Writing – review & editing. **Carolina Sales Vieira:** Investigation, Writing – review & editing. **Mariane Nunes Nadai:** Conceptualization, Methodology, Supervision. **Sérgio Henrique Pires Okano:** Investigation, Writing – review & editing. **Silvio Antônio Franceschini:** Investigation, Writing – review & editing. **Lúcia Alves da Silva Lara:** Conceptualization, Methodology, Supervision.

## Conflicts of interest

The authors declare no conflicts of interest.
